# DDPD 1.0: a manually curated and standardized database of digital properties of approved drugs for drug-likeness evaluation and drug development

**DOI:** 10.1093/database/baab083

**Published:** 2022-02-07

**Authors:** Qiang Li, Shiyong Ma, Xuelu Zhang, Zhaoyu Zhai, Lu Zhou, Haodong Tao, Yachen Wang, Jianbo Pan

**Affiliations:** Center for Novel Target and Therapeutic Intervention, Institute of Life Sciences, Chongqing Medical University, 1 Yixueyuan Road, Yuzhong District, Chongqing 400016, China; Center for Novel Target and Therapeutic Intervention, Institute of Life Sciences, Chongqing Medical University, 1 Yixueyuan Road, Yuzhong District, Chongqing 400016, China; Center for Novel Target and Therapeutic Intervention, Institute of Life Sciences, Chongqing Medical University, 1 Yixueyuan Road, Yuzhong District, Chongqing 400016, China; Center for Novel Target and Therapeutic Intervention, Institute of Life Sciences, Chongqing Medical University, 1 Yixueyuan Road, Yuzhong District, Chongqing 400016, China; Center for Novel Target and Therapeutic Intervention, Institute of Life Sciences, Chongqing Medical University, 1 Yixueyuan Road, Yuzhong District, Chongqing 400016, China; Center for Novel Target and Therapeutic Intervention, Institute of Life Sciences, Chongqing Medical University, 1 Yixueyuan Road, Yuzhong District, Chongqing 400016, China; Center for Novel Target and Therapeutic Intervention, Institute of Life Sciences, Chongqing Medical University, 1 Yixueyuan Road, Yuzhong District, Chongqing 400016, China; Center for Novel Target and Therapeutic Intervention, Institute of Life Sciences, Chongqing Medical University, 1 Yixueyuan Road, Yuzhong District, Chongqing 400016, China

## Abstract

Drug-likeness is a vital consideration when selecting compounds in the early stage of drug discovery. A series of drug-like properties are needed to predict the drug-likeness of a given compound and provide useful guidelines to increase the likelihood of converting lead compounds into drugs. Experimental physicochemical properties, pharmacokinetic/toxicokinetic properties and maximum dosages of approved small-molecule drugs from multiple text-based unstructured data resources have been manually assembled, curated, further digitized and processed into structured data, which are deposited in the Database of Digital Properties of approved Drugs (DDPD). DDPD 1.0 contains 30 212 drug property entries, including 2250 approved drugs and 32 properties, in a standardized value/unit format. Moreover, two analysis tools are provided to examine the drug-likeness features of given molecules based on the collected property data of approved drugs. Additionally, three case studies are presented to demonstrate how users can utilize the database. We believe that this database will be a valuable resource for the drug discovery and development field.

**Database URL**: http://www.inbirg.com/ddpd

## Introduction

In the early stage of the drug design/discovery process, drug-likeness is a vital consideration when selecting compounds. Drug-likeness evaluation can provide useful guidelines to increase the likelihood of converting lead compounds into drugs. For example, to guarantee that a drug can function effectively as a clinical treatment, an appropriate concentration at the target site must been ensured. Although dosage and administration are obviously two important factors, the concentration of a drug also depends on its absorption, distribution, binding or local accumulation in the tissue, the degree and rate of biotransformation, excretion and other variables (1). Therefore, physicochemical property, pharmacokinetic (PK), toxicological and maximum dosage studies of drugs are important components of drug-likeness evaluation. Statistical and computational modeling of the molecular dynamic regulation of drugs within the body is of great theoretical value and practical significance for broad usage. For instance, a number of *in silico* approaches including machine-learning methods have been successfully applied in the evaluation of drugs (2), virtual screening (3), drug property prediction (4, 5), ADMET prediction (6, 7), drug target prediction (8, 9), pharmaceutical dosage form improvement (10) and design and optimization of dosing regimens (11).

To facilitate the development of drug-likeness evaluation methods, high-quality and comprehensive datasets of drug-likeness properties are essential. However, most high-quality datasets are not publicly accessible, primarily because of commercial conflicts of interest. In particular, regarding the PK, toxicity and dose information of drugs, most of the related data are unorganized in different databases or sparsely scattered in the text of relevant references. Several databases contain property values for numbers of drugs. For example, PK/DB, which has been unavailable for several years despite being published in 2008, manages 2973 property values involving 1203 compounds and 8 PK features (12). PK-DB is a database containing 512 PK studies from clinical trials, and it provides automatic calculation of eight PK parameters from the data (13). However, the data contained in the available databases are limited. Most entries only have several PK properties, and the databases suffer from high rates of missing values. Furthermore, some databases are not specifically designed for drug screening, comparison and analysis based on property data. Therefore, the lack of a comprehensive standardized data portal for digital drug properties has been a major obstacle in drug-likeness prediction and drug development.

In this study, we introduced a user-friendly database named Database of Digital Properties of approved Drugs (DDPD) that provides a centralized data portal of manually curated and standardized digital drug properties. DDPD includes the experimental physicochemical properties, PK/toxicokinetic properties and maximum dosages of approved small-molecule drugs. Furthermore, downstream statistical analyses based on the collected dataset were developed to provide confidence intervals for the drug-likeliness of given small molecules using appropriately chosen statistical assumptions and models. Driven by the need for *in silico* modeling of drug properties, the development of DDPD is paving the road for effective virtual screening and small molecule drug design and ultimately expected to contribute to accelerating drug discovery and development.

## Materials and methods

### Data collection and curation

In this study, six authoritative resources of drugs were explored, including DrugBank (14), T3DB (15), ATSDR (https://www.atsdrcdc.gov/), PDR (https://www.pdrnet/), ‘*The Pharmacological Basis of Therapeutics*’ (1) and a human intravenous PK dataset (16). Drug-like property related features such as physicochemical properties (e.g. log P, water solubility), PK properties (e.g. bioavailability, biological half-life, protein-binding rate), toxicity information and dosage data were extracted from these resources. It is notable that only experimental property values and not predictive values were extracted.

Specifically, drug structures represented using the simplified molecular-input line-entry system (SMILES), physicochemical property and PK data of the small-molecule drugs were extracted manually through reading the text of the drug descriptions in DrugBank. In T3DB and ATSDR, the toxicity information of small-molecule drugs was extracted. The maximum dosage information of the corresponding commercial drugs was obtained from the PDR database. In the appendix of *The Pharmacological Basis of Therapeutics* and the human intravenous PK dataset, drug properties including bioavailability, urinary excretion, serum protein binding rate, clearance rate, apparent volume of distribution, biological half-life, peak time and peak density were manually obtained via text reading, and the eight drug properties from this appendix were treated as supplementary to the data from DrugBank. The origins of the properties of drugs were also recorded in the DDPD.

Because the data were collected from multiple heterogeneous and unstructured databases, several data preprocessing steps were implemented to increase data integrity for downstream data analytics either performed by users or provided by the in-house developed services. Therefore, normalization of the assembled data, including the standardization and unification of units, was performed. Moreover, the characteristics of individuals including age, weight, gender, cigarette smoking habits, genomic variants and diseases and routes of drug administration affect the property values. Consequently, certain parameters of one drug might have multiple values because of differences in the conditions for different individuals. Therefore, it was necessary to digitize the recordings of these factors for each data entry in the database. The aim was to reduce confounding and improve the quality of knowledge distilled from data analyses.

### Database implementation

Techniques including Ngnix and uwsgi were adopted as the web containers for services supply. Django and MySql were used for back-end data interchange, and Bootstrap4 and Jquery were applied for front-end visualization. Statistical analyses were performed using python packages including Pandas (1.0.5), NumPy (1.18.5) and SciPy (1.4.1). Data analyses were visualized using several visualization approaches including Bar Chart and Radar Chart.

## Results

### Data retrieval

**Figure 1.**
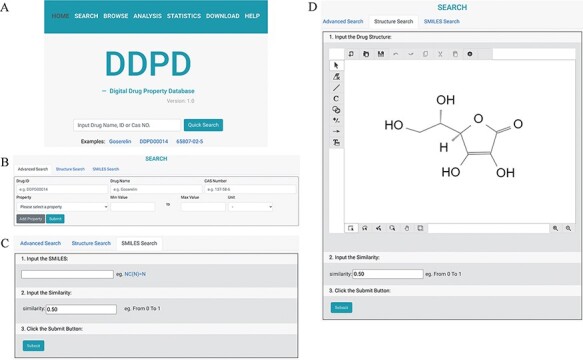
The search pages of DDPD: (A) On the home page, users can conduct simple search via drug name, DDPD ID or CAS number. (B, C, D) On the search page, users can perform more advanced searches via one or more drug property values, SMILES or structure. (D) In Structure Search tab, users can draw chemical structures using the developed drawing tool.

**Figure 2.**
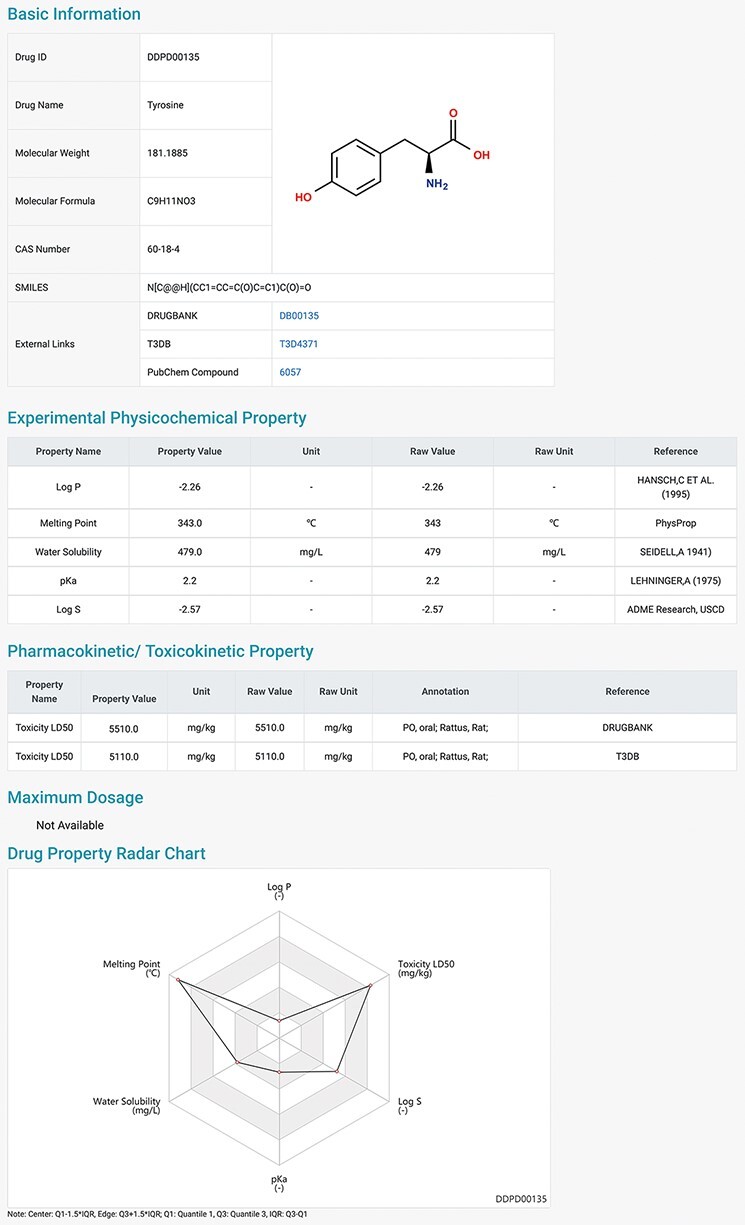
Detailed information page of a drug example. Users can view detailed information of the selected drug, including basic information, physicochemical properties, pharmacokinetic and toxicokinetic properties, as well as maximum dosages. A radar chart which displays the values of all the properties of the selected drug is shown at the bottom.

DDPD can be accessed at http://www.inbirg.com/ddpd. Multiple data-retrieval methods were developed for accessing DDPD and summarized in a schematic view (Figure 1). A quick search by gene name, gene ID or CAS number is provided on the *HOME* page (Figure 1A). On the *SEARCH* page, users can perform three other types of searches in an effort to improve the usability of the database. For the *Advanced Search*, users can retrieve the property information of drugs more accurately by inputting the drug ID/name and the intervals of its property values (Figure 1B). For the *SMILES Search*, drugs with similar SMILES strings to the input will be obtained using the regular string comparison method of RDKit (https://www.rdkit.org/) (Figure 1C). For the *Structure Search* (Figure 1D), using structure drawing board Kekule.js (17), a molecule structure of interest must be drawn manually first and will then be automatically converted into SMILES string, and drugs with similar SMILES strings would be obtained subsequently. The SMILES/structure-based searches allow users to retrieve drugs with similar sub-structures to facilitate structure–property relationship analysis. All performed searches will be linked to a *Search Result Page* on which all matched drugs are listed and presented for download. Users can click on the found drug ID, and they can be redirected to a page displaying detailed information about the drug (Figure 2), which contains the *Basic Information, Experimental Physicochemical Property, PK/Toxicokinetic Property* and *Maximum Dosage*. As unique features of DDPD, annotations (e.g. routes, populations) and factors (e.g. foods, ages) are also provided for many of the property entries. A *Drug Property Radar Chart* is provided for each drug to display the values of properties along their own property axis within the dataset (Figure 2). On the *BROWSE* page, the entire list of the collected drugs can be reviewed by drug name/ID in alphabetical order. In addition, the entire database can be downloaded from the *DOWNLOAD* page. The *HELP* page walks users through essential definitions of the terminologies on the website and explanations of the basic functions of each webpage.

### The statistics of DDPD

**Figure 3.**
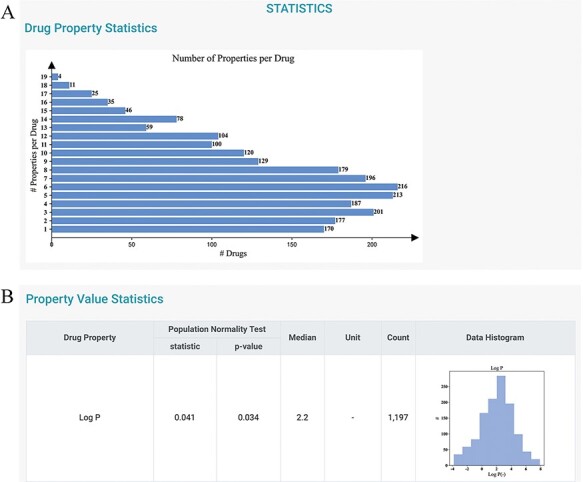
Statistics page of DDPD. (A) Barplot shows the number of properties per drug. (B) Property value statistics such as histogram for log P is provided. Log P of all drugs in our database is to some degree normally distributed with a median of 2.2 (normality test *P*-value = 0.034).

To gain further information about the distributions of the drug properties, users can consult the *STATISTICS* and *HOME* pages. The current version of DDPD contains 2250 approved drugs and 32 properties (i.e. 7 physicochemical properties, 18 PK/toxicokinetic properties and 7 maximum dosages of different populations). A total of 30 212 (18 011 non-redundant) drug–digital properties including 4443 physicochemical properties, 18 016 PK/toxicokinetic properties and 7753 maximum dosage values with standardized value/unit format are deposited. On the *STATISTICS* page, statistics of the number of properties per drug are given (Figure 3A); the statistics of the properties are also provided to indicate the distributions of drug properties, and they can be potentially useful for statisticians to construct more appropriate parametric hypotheses (Figure 3B).

### Drug property analysis

**Figure 4.**
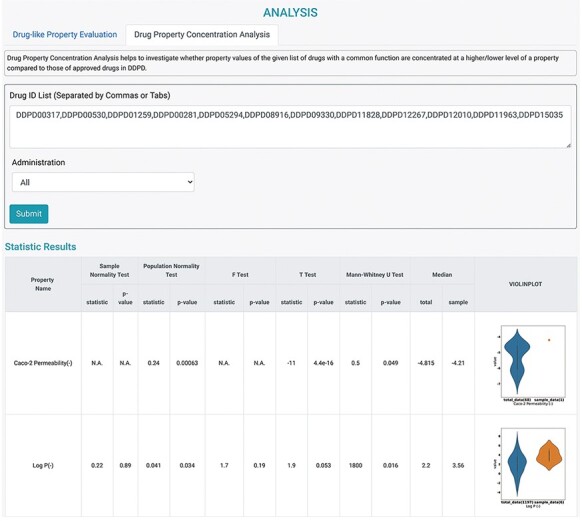
Analysis tools of DDPD. Using the Drug Property Concentration Analysis tab of the Analysis page, users can query a list of drugs via DDPD ID and visualize the property value distribution of the query list via violin plots. The blue violin plots represent the property value distribution of all the approved drugs in our database. The orange plots represent the distribution of the queried compounds. For the queried drugs for caco-2 permeability, only one data entry is found, therefore, one orange dot is shown. Additionally, the pvalues of the selected statistical tests are calculated and listed in the respective columns.

It is assumed that the property values of the approved drugs collectively contribute to a distribution that is informative for the eligibility of being a usable drug. More specifically, for the given investigated molecules, the confidence that a molecule can become an effective drug increases as the drug property value approaches the center of the distribution. Therefore, several statistical models were adopted to implicitly estimate the confidence of drug-likeness for the given molecules via univariate analysis. Two analysis tools were designed according to the assumptions, and they could be used on the *ANALYSIS* page (Figure 4). First, Drug-like Property Evaluation was designed to check whether the property values of the investigated drug(s) are significantly different from those of the approved drugs in DDPD. Radar chart is provided to display median values of the given property values along their own property axis within the dataset. The *P*-values calculated using Student’s *t*-test and the Mann–Whitney *U* test are given to measure the significance of the differences between the sample mean and population mean (the whole dataset) of the input drug property. Furthermore, violin plots are presented to visualize the distributions of the queried features of the whole dataset and input. Second, Drug Property Concentration Analysis was developed to investigate whether the property values of the given list of drugs with a common function are concentrated at a higher/lower level compared with those of the approved drugs in this database (Figure 4). Radar chart, calculated *P*-values and violin plots are also provided.

### Case studies

Three case studies investigating different drug properties are presented to demonstrate how users can utilize the database.

#### Case study 1: Esmolol

Esmolol, a cardio selective beta blocker, is used for the short-term control of ventricular rate and heart rate in various types of tachycardia (18). By searching (or browsing) through DDPD, users can get the property values of esmolol as follows:

- On the search bar of Home Page, type in ‘esmolol’ and press enter.
- The search result will display esmolol as the only result along with its DDPD Drug ID, CAS number, etc.
- Clicking on the drug ID will redirect users to the detailed information page. By scrolling to the bottom, users can view the drug property radar chart of esmolol.

**Figure 5.**
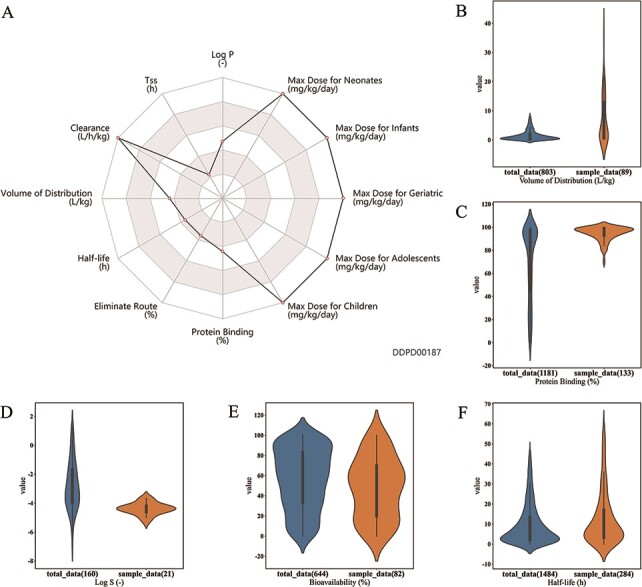
Case studies. (A) Case study 1. Radar chart for properties of esmolol. Property values related to maximum dosage and clearance are shown to be at the maximums of the corresponding ranges. (B, C, D, E). Case Study 2. Drugs with log P value greater than 4 are selected as the sample group; as shown, property value distributions for these drugs (orange plots) are significantly different from those of all approved small molecule drugs (blue plots): (B) Volume of distribution is significantly higher for the sample group (median 3.4 > 0.85, *P*-value = 3.1e–11); (C) Protein binding is significantly higher for the sample group (median 97 > 85, *P*-value = 9.8e-21); (D) Log S is significantly lower for the sample group (median −4.39 < −3.03, *P*-value= 1.6e-7); and (E) bioavailability is significantly lower for the sample group (median 50 < 60, *P*-value= 0.0061). (F) Case study 3. Half-life of 373 albumin related drugs is compared with half-life of all approved small molecule drugs. Half-life is significantly higher for albumin related drugs (median 7.4 > 6.04, *P*-value = 0.0003). All *P*-values are calculated using Mann–Whitney *U* Test performed by the developed analysis tool.

As shown on the radar chart, esmolol has relatively high clearance and maximum dosages for various demographic groups (Figure 5A). Furthermore, it also has relatively short time to reach steady state, which indicates good absorption (18). Esmolol can maintain a steady state within 5 min of infusion and can be effectively cleared after the discontinuation of the infusion. (18) In other words, esmolol can be quickly cleared in the human body and safely administered in high tolerated dose as demonstrated in Figure 5A. This study indicates that DDPD can help users improve overall understanding of a drug’s PK behavior.

#### Case Study 2: Characteristic of drugs with high lipophilicity

The common properties of drugs with high lipophilicity have been investigated in this case study, demonstrating the usage of the advanced search function and the ‘Drug Property Concentration Analysis’ tool.

- First, based on the statistics in DDPD, log P, a measure of lipophilicity, exhibits normal distribution (Figure 3B).
- In the advanced search, select log P > 4 in this case, and 232 drugs can be found and downloaded (Supplementary Table S1).
- In the next step, input the extracted Drug IDs into the text frame in ‘Drug Property Concentration Analysis’ on the analysis page. By clicking the submit button, the results show that these drugs are significantly concentrated in a higher volume of distribution (19) (Figure 5B), higher protein binding rate (20) (Figure 5C), lower log S (21) (Figure 5D) and lower bioavailability (22) (Figure 5E), compared to all approved drugs in the database.

This analysis reveals the relationship between lipophilicity and other drug properties, and it provides clues for chemical property research and predictive modeling.

#### Case study 3: Analysis of serum albumin related drugs

In this case study, property value distributions of serum albumin related drugs have been investigated. The workflow is described below and the use of DrugBank is also included.

- First, select 373 approved small molecule drugs from serum albumin’s drug relations table in DrugBank.
- On the Analysis page, under the Drug Property Concentration Analysis tab, enter the converted IDs (replace ‘DB’ to ‘DDPD’) for all 373 drugs in the Drug ID List query box (Supplementary Table S2).
- Select ‘All’ for the Administration option underneath the query box and click submit.
- Results show a radar chart summarizing properties values for the 373 searched drugs. Below the radar chart is the property value comparison between the 373 searched drugs and all approved small molecule drugs.

Half-life is the most significantly enriched property of these drugs. The selected 373 drugs have a median half-life of 7.4, which is significantly higher than the median (6.04) of the half-life of all approved small molecule drugs (*P*-value = 0.0003, Figure 5F). It has been reported that albumin could extend the circulatory half-life of drugs (23, 24). As the main protein of plasma, serum albumin has a good binding capacity for drugs. In other words, according to this finding, it is likely that certain drugs can be modified or engineered to bind to serum albumin so as to prolong half-life and improve drug-likeness (25).

## Discussion

The complexity of evaluating drug-likeness of given molecules should never be underestimated, and practical methods are urgently demanded for inferring the drug-likeness of investigated molecules. For the drug-likeness evaluation in this study, we assumed that the quantitative principles of molecular properties including physicochemical and PK properties must be obeyed to permit the molecules to reach the drug target at a sufficient concentration for a sufficient duration. Traditional methods for drug-likeness evaluation such as Lipinski’s Rule of Five (26) provide useful information and suggest that molecules disobeying quantitative rules of the drug properties would not function effectively in biological systems However, because simple models may not always be accurate, this type of analysis is inherently limited, and it should be used with caution. An increasing number of compounds disobeying Lipinski’s Rule of Five are being approved by the FDA (27). It has also been proven that by combining more descriptors of molecules, the identification of drug-likeness molecules can be substantially improved using machine learning (28) compared with using Lipinski’s Rule of Five. Therefore, DDPD was developed to provide high-quality digital properties including the experimental physicochemical properties, PK/toxicokinetic properties and maximum dosages of approved small molecule drugs, and it might emerge as a useful resource for researchers to mine more accurate patterns for inferring drug-likeness. Popular databases, such as DrugBank (14), contain large amount of information about drugs and offer broad scope, comprehensive referencing and detailed data descriptions. Yet, most numerical values of drug properties in DrugBank are embedded in textual descriptions, which makes the values of drug properties hard to be indexed and extracted for data analysis. Property values in DDPD have been manually extracted, standardized and annotated to enable accurate statistical comparisons and modeling. Moreover, the structured data can be easily searched, browsed, extracted and downloaded through a user-friendly interactive interface in DDPD. Other databases such as PK/DB and PK-DB primarily focuses on curating PK properties (12, 13). In addition to larger number of PK properties (32, 8, 8 for DDPD, PK/DB, PK-DB, respectively), DDPD also curates toxicokinetics, physicochemical properties, as well as maximum dosage properties. Additionally, PK-DB mainly contains time-course measurements in clinical studies in semi-structured format, however, DDPD deposits drug-centered property values in structured format to make the process of drug property analysis streamlined. Furthermore, DDPD provides built-in analysis tools as a preparatory application based on the collected digital drug properties, which could provide information for the drug-likeness of the given molecules.

Despite the complexity of drug-like mechanisms, it is promising that the developed tools could be informative for researchers to direct their studies and contribute to shortening the path for drug screening and design. *In silico* approaches, including machine-learning methods, can be applied for drug evaluation (2) and drug property prediction. (4) Therefore, in future versions of DDPD, models and methods of predictions of drug features based on machine learning and/or big data techniques will be implemented. Usability of DDPD will be continuously improved, so that a streamlined prediction of drug-likeness can be easily achieved by users. Further plans are in place to regularly updating the database every 6 months with newly published data. Moreover, drugs in clinical trials or those withdrawn from development are scheduled to be incorporated to expand the datasets. In addition, drug sensitivity data for cell lines and model organisms will also be collected.

In summary, DDPD is a remarkable comprehensive public repository presently available for obtaining the standardized experimental property values of approved drugs. DDPD both provides high-quality manually curated digital drug properties and offers advanced computational analysis services. This database is expected to become a public hub for *in silico* drug-likeness assessment. We believe that this database will be a valuable resource for the drug discovery and development field.

## Supplementary Material

baab083_Supp
