# Correction to: DDPD 1.0: a manually curated and standardized database of digital properties of approved drugs for drug-likeness evaluation and drug development

**DOI:** 10.1093/database/baac016

**Published:** 2022-03-23

**Authors:** Qiang Li, Shiyong Ma, Xuelu Zhang, Zhaoyu Zhai, Lu Zhou, Haodong Tao, Yachen Wang, Jianbo Pan

**Affiliations:** Center for Novel Target and Therapeutic Intervention, Institute of Life Sciences, Chongqing Medical University, 1 Yixueyuan Road, Yuzhong District, Chongqing 400016, China; Center for Novel Target and Therapeutic Intervention, Institute of Life Sciences, Chongqing Medical University, 1 Yixueyuan Road, Yuzhong District, Chongqing 400016, China; Center for Novel Target and Therapeutic Intervention, Institute of Life Sciences, Chongqing Medical University, 1 Yixueyuan Road, Yuzhong District, Chongqing 400016, China; Center for Novel Target and Therapeutic Intervention, Institute of Life Sciences, Chongqing Medical University, 1 Yixueyuan Road, Yuzhong District, Chongqing 400016, China; Center for Novel Target and Therapeutic Intervention, Institute of Life Sciences, Chongqing Medical University, 1 Yixueyuan Road, Yuzhong District, Chongqing 400016, China; Center for Novel Target and Therapeutic Intervention, Institute of Life Sciences, Chongqing Medical University, 1 Yixueyuan Road, Yuzhong District, Chongqing 400016, China; Center for Novel Target and Therapeutic Intervention, Institute of Life Sciences, Chongqing Medical University, 1 Yixueyuan Road, Yuzhong District, Chongqing 400016, China; Center for Novel Target and Therapeutic Intervention, Institute of Life Sciences, Chongqing Medical University, 1 Yixueyuan Road, Yuzhong District, Chongqing 400016, China

In the originally published PDF version of the manuscript, there was an error in the Citation Details. These should read: ‘Database (2022) Vol. 2022:’ instead of ‘Database (2021) Vol. 2021:’.

